# Research on Dynamic Spectrum Sharing in the Internet of Vehicles Based on Blockchain and Game Theory

**DOI:** 10.3390/s26041190

**Published:** 2026-02-12

**Authors:** Xianhao Shen, Mingze Li, Jiazhi Yang, Jinsheng Yi

**Affiliations:** 1 College of Physics and Electronic Information Engineering, Guilin University of Technology, Guilin 541006, China; 2008067@glut.edu.cn; 2 College of Computer Science and Engineering, Guilin University of Technology, Guilin 541006, China; 1020231206@glut.edu.cn; 3 Guangxi Key Laboratory of Special Engineering Equipment and Control, Guilin University of Aerospace Technology, Guilin 541004, China; jiazhi.yang@guat.edu.cn

**Keywords:** Internet of Vehicles, blockchain, Stackelberg game, consensus mechanism, dynamic spectrum sharing

## Abstract

With the rapid development of the Internet of Vehicles (IoV), the explosive growth of data traffic within the system has led to a surge in demand for spectrum resources. However, the strict limitations on spectrum supply make the construction of an efficient and reasonable resource allocation scheme crucial for IoV. To maximize social benefits and improve security in the resource allocation process under IoV spectrum scarcity, this paper proposes a dynamic spectrum allocation (DSA) scheme based on a consortium blockchain framework. In this scheme, we design a demand-based vehicle priority classification method and propose a novel hybrid consensus mechanism—PhDPoR—which integrates practical byzantine fault tolerance (PBFT) and Hierarchical Delegated Proof of Reputation. Furthermore, we construct a multi-leader, multi-follower (MLMF) Stackelberg game model and utilize smart contracts to implement an immutable on-chain record of spectrum resource allocation, thereby deriving the optimal spectrum pricing and purchase strategy. Experimental results show that the proposed scheme not only effectively optimizes the utility of both supply and demand sides and improves overall social benefits while ensuring efficiency, but also significantly outperforms baseline algorithms in identifying and mitigating malicious nodes, thus verifying its feasibility and application value in complex IoV environments.

## 1. Introduction

Currently, nations worldwide are vigorously developing smart city transportation systems, with Internet of Vehicles communication at their core. Therefore, the Internet of Vehicles (IoV) represents an inevitable trend for the future [1], garnering significant interest from researchers globally [2]. However, due to the dramatic increase [3] in the number of vehicles and intelligent devices, IoV communication is required to process massive amounts of sensitive data in real time, leading to an exponential growth in the demand for spectrum resources. To guarantee various strict QoS requirements and adapt to the complex and highly dynamic IoV environment, designing efficient spectrum resource allocation schemes is paramount; thus, we introduce the MLMF Stackelberg game for dynamic spectrum resource allocation. In this game model, there are multiple leaders and followers; the leaders make decisions first, and the followers subsequently make rational responses based on the leaders’ decisions. In the context of IoV spectrum allocation, the seller base stations assume the role of leaders, while the buyer vehicles serve as followers.

Given the high heterogeneity of data service demands in the IoV environment, distinct vehicle types exhibit significant differences in Quality of Service requirements. In particular, for emergency vehicles such as ambulances executing time-critical tasks, failure to prioritize their communication needs may lead to severe consequences. To address this, we propose a priority-based vehicle classification mechanism designed to optimize the interaction process. By quantifying the urgency factor of demands and employing an asymmetric pricing strategy, this mechanism incentivizes base stations to prioritize the scheduling of high-value tasks. This approach not only ensures real-time responsiveness in dynamic resource allocation but also effectively maximizes the overall social welfare of the system.

However, due to potential security vulnerabilities [4] in the IoV environment, vehicle users may be subject to attacks from external or internal adversaries [5]. Therefore, ensuring information security in the multi-party coexistence environment of IoV is crucial for its stable operation. To this end, we introduce blockchain, a distributed ledger technology [6] characterized by transparency, anonymity, decentralization, and immutability [7,8]. The core of blockchain is the problem known as mining; to guarantee the integrity and validity of transactions, a group of participants called miners must complete a proof of work challenge. In traditional public and permissionless blockchains, the consensus phase is executed by all miners, incurring high computational cost [9]. To alleviate this issue, in this paper, we select a consortium blockchain to implement secure spectrum trading between spectrum requesters and providers, executing the consensus process on pre-selected nodes with moderate collaboration costs.

Utilizing blockchain provides a secure and reliable environment [10] for communication between IoV entities and, in combination with smart contracts, automates the spectrum resource allocation and management process. The main contributions of this paper are as follows:

1. A classification method based on vehicle demand urgency is proposed. The higher the vehicle demand urgency is, the higher the vehicle priority is. The priority of vehicle demand plays a role in the consensus mechanism PhDPoR with voting rights.
2. The PhDPoR consensus algorithm is proposed, which achieves the optimal solution for spectrum resource allocation in terms of economic efficiency while ensuring consensus determinism.
3. We model the interaction between buyers and sellers in this scenario as an MLMF Stackelberg game, use asymmetric pricing to conduct dynamic spectrum trading, optimize the allocation of spectrum resources, and prove the existence of Stackelberg equilibrium.
4. We introduce reputation evaluation into vehicles and base stations, and select master nodes and verifiers according to reputation. Reputation evaluation plays an important role in resource allocation and block verification tasks.

## 2. Related Work

In order to overcome the limitations of traditional methods, researchers propose to apply blockchain in the allocation and management of spectrum resources to solve the problem of privacy and security. In reference [11], the author proposes a resource sharing system among the major operators in the alliance chain, which connects the users who need to rent spectrum, effectively improves the spectrum utilization of the operators, and reliably saves the spectrum transactions to the block. Due to its advantages of decentralization, anonymity, and trust, blockchain has been applied to various scenarios of in-vehicle networks [12]. Reference [13] proposes a trust management scheme based on blockchain smart contract for IoV security and privacy threats, and introduces fragmentation technology to improve the scalability of the system. Liu et al. [14] proposed a smart contract-based double auction framework, in which the entire transaction process is executed automatically, ensuring the fairness and reliability of transactions. Xue et al. [15] proposed a spectrum trading scheme based on blockchain to solve the problems of low spectrum utilization and high transaction delay under the traditional method. In addition, reference [16] proposes an interference-based consensus mechanism and block transaction verification mechanism for the potential harmful interference problem in spectrum sharing. Li et al. [17] designed an alliance blockchain to realize trusted dynamic spectrum sharing for the Internet of Vehicles, and proposed an improved asynchronous Byzantine fault-tolerant algorithm to solve this problem considering the signal instability in this scenario. Reference [18] designs a blockchain trust management system based on the improved Byzantine fault-tolerant consensus mechanism of joint proof of work (PoW), which improves the scalability of the system, but the throughput of the blockchain system is low. In PoW, miners compete for leaders by solving mathematical puzzles, which consume more computing resources and time. In order to solve this problem, Ethereum proposed a proof of stake (PoS) consensus mechanism, which randomly selects miners according to the rights and interests of nodes in the system [19]. However, in the PoS consensus process, centralization may occur because some stakeholders control the central process. Later, researchers proposed a delegated PoS (DPoS) consensus mechanism. This mechanism processes transactions faster than PoS [20]. However, these mechanisms are not entirely desirable, for example, in PoS and DPoS consensus mechanisms, which may lead to malicious mining attacks by high-risk nodes. In addition, a manner of calculating a reputation value by using a subjective trust model and then selecting a verifier according to the reputation value becomes a PoR. In 1999, Castro et al. also introduced PBFT [21], which is now used in Hyperledger. Nodes participating in consensus must exchange information with other nodes to ensure the network security of PBFT. Reputation-based consensus mechanisms [22] have been shown to effectively reduce the communication complexity of vehicle-to-everything (V2X) networks and optimize validator selection. However, this research focuses on balancing system energy consumption and utility through algorithm optimization, neglecting the inherent non-cooperative game behavior and pricing equilibrium challenges of multi-party market entities in dynamic spectrum trading.

The literature [23] divides the IoV data market into three parties and proposes a pricing mechanism based on Stackelberg game to solve the conflicts of interest among the three parties. However, the model assumes that the market information is idealized and cannot fully adapt to the real-time dynamic market environment. Feng et al. [24] found that there will be a contradiction with the interference behavior in the communication countermeasure, and proposed a hierarchical Stackleberg game model based on the spectrum compensation mechanism (SCM) to solve this problem. In addition, some scholars have studied the UAV-assisted resource allocation by introducing the Stackelberg game, for example, Hisseini and his team [25] used the characteristics of UAV, such as flexibility and rapid deployment, and combined with the game to assist the vehicle communication network to allocate radio resources reasonably. Qiu et al. [9] predicted that UAV will become an important part of the cellular network in the future. Considering the security risks of UAV network communication, in view of this problem, blockchain technology and the Stackelberg game are introduced to realize secure spectrum allocation for UAV network communication. Reference [26] studies how to deploy small base stations inside buildings to support dynamic spectrum sharing technology in mobile communications, while reference [27] describes spectrum sharing and dynamic spectrum allocation in cognitive radio network scenarios, focusing on allocating unused spectrum to secondary users.

## 3. System Model

### 3.1. Model Architecture

**Figure 1:**
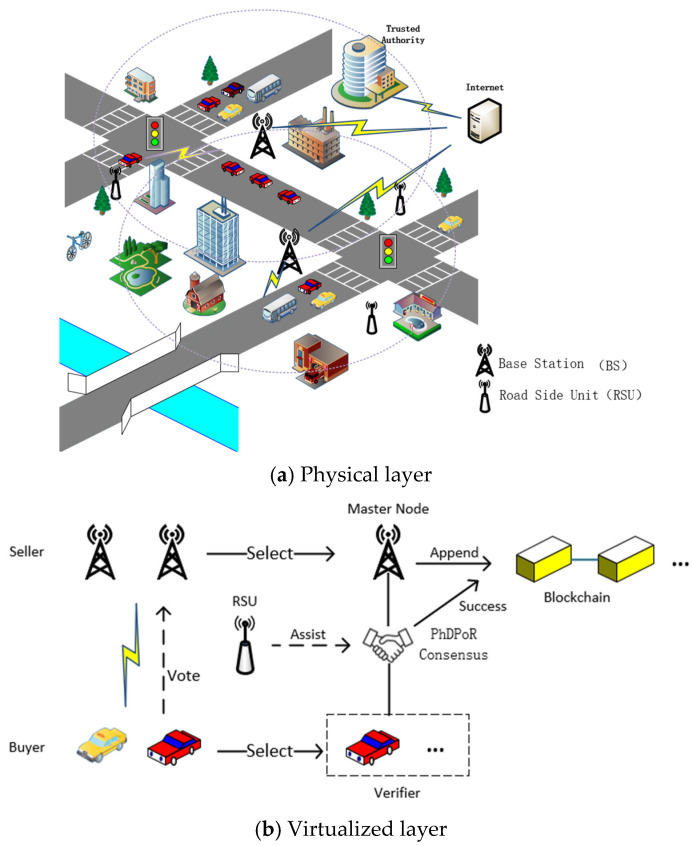
System model diagram in the Internet of Vehicles scenario.

The system model constructed in this section is shown in Figure 1. This scenario consists of base stations, vehicle users, and roadside units (RSUs). The base station, as an authorized infrastructure provider for the mobile network operator, is responsible for providing critical spectrum resources to vehicles. This critical public infrastructure is supervised by relevant regulatory agencies (such as the government). The core participating entities in the system are the base stations and different types of vehicles. Vehicles interact with base stations via vehicle-to-infrastructure (V2I) links through their onboard communication units. Interconnection can also be established between different base stations. In the proposed scenario, multiple base stations coexist in the same geographical area and each maintains its own network coverage. When a vehicle has a communication need, it can dynamically associate it with the optimal base station based on its actual service requirements. In addition, the system deploys several roadside units (RSUs) as auxiliary nodes to access the base station’s network infrastructure. It is worth noting that this article mainly focuses on the service channel, while emergency safety messages (such as emergency braking signals) are transmitted by a dedicated control channel.

The system architecture is mainly divided into a physical layer and a virtual layer. In the physical layer, each participant must be authenticated by a Trust Authority (TA). Upon successful authentication, they receive their own public and private keys, as well as encryption and decryption certificates, becoming a uniquely authorized entity and acquiring initial reputation. When a vehicle requests spectrum, it initiates a request to the base station. The buyer and seller interact through an MLMF Stackelberg game to achieve equilibrium. Subsequently, smart contracts are used to execute block verification, resource allocation, and updates to vehicle and base station reputation information. In the virtual layer, after consensus begins, high-reputation vehicles are selected as blockchain verification nodes based on a reputation assessment mechanism. Roadside units are also introduced as auxiliary consensus nodes to ensure the security and reliability of the resource allocation process. In the PhDPoR consensus process, participating nodes record data interaction information during vehicle–base station communication. Specific details about the blockchain consensus mechanism will be elaborated in Section 5.

### 3.2. Vehicle Demand Urgency Index

In the Internet of Vehicles (IoV) environment, the functional attributes of vehicles exhibit significant diversity, leading to differentiated resource requirements across different vehicle types. To effectively adapt to and meet these heterogeneous service demands, this paper classifies vehicles based on their purpose and the nature of their needs. Furthermore, based on the social benefits embodied by different vehicles, vehicle demand priorities are divided into four levels and quantified as vehicle demand urgency indicators. Vehicle demand priorities will play a major role in resource allocation and blockchain consensus stages. This classification method achieves a comprehensive determination of vehicle demand priorities, as detailed in Table 1.

When a vehicle encounters an emergency, it is allowed to temporarily increase its priority to secure necessary spectrum resources. However, during the block verification phase, consensus nodes strictly monitor the use of this privilege. Specifically, this monitoring is implemented through two verification rules in the smart contract. First, consensus nodes check the historical ledger to ensure that the frequency of high-priority claims by a single vehicle does not exceed a statistical threshold. Second, consensus nodes verify whether the data type of the request matches the emergency type. If a vehicle violates these rules, it will be identified as a malicious node, and its reputation value will be significantly reduced to prevent resource monopoly.

In addition, we incorporate reputation opinions into the blockchain, which can provide lasting and public evidence when disputes arise. When the vehicle uploads the transaction data for processing, the vehicle can evaluate the base station and the nodes participating in the consensus process. The reputation opinion is dynamically updated. It is updated by combining the new opinion of the vehicle with the existing reputation opinion of the entity in the blockchain. The new opinion of the vehicle produces a digital signature and uploads the signature to other nodes nearby. By updating the reputation opinion, the whole resource allocation process can rely on the latest reputation information to make decisions. At the same time, the reputation value information will also be used as an important data indicator for the verifier node selection and block verification process.

### 3.3. Smart Contract

When a blockchain node receives a spectrum resource request, the system initiates an MLMF Stackelberg game to coordinate supply and demand and pricing strategies under given constraints. As the leader–follower strategy gradually converges, the game reaches equilibrium, and the smart contract is triggered. In the system architecture presented in this paper, the smart contract performs three core functions: first, it completes the consensus process and records key decisions to ensure traceability and tamper-proof results; second, it dynamically updates the node’s reputation based on the results of transactions and interactions to incentivize honest behavior and suppress opportunistic behavior; finally, according to the equilibrium scheme, spectrum resources are automatically allocated and settled, thereby achieving consistency and verifiability in strategy execution and resource allocation on the chain, the details are shown in Table 2.

## 4. Formulate the Problem

### 4.1. Profit Model

In the proposed model, buyers and sellers realize their respective gains through resource exchange, and both parties aim to maximize their individual interests. To this end, we employ the MLMF Stackelberg game model to optimize the profits of both buyers and sellers.

Assume that, in this scenario, the base station participating in spectrum allocation holds a total spectrum resource of $P^{l}$, where l=1,2,…,K indexes different base stations. There are A vehicles that need to obtain spectrum resources from this base station, and each vehicle needs to purchase bandwidth $V_{i}$, for i=1,2,…,A. To meet the bandwidth requirements of vehicles with different priority levels, the following condition must be satisfied:

(1) $$\sum_{N_{Q}}V_{i,Q}^{l}\leq P^{l}-\left(\sum_{N_{Q-1}}V_{i,Q-1}^{l}+\sum_{N_{Q-2}}V_{i,Q-2}^{l}+\cdots +\sum_{N_{1}}V_{i,1}^{l}\right)$$

where Q∈{1,2,3,4} denotes the vehicle priority level, $N_{Q}$ is the number of vehicles with priority Q, and $V_{i,Q}^{l}$ represents the spectrum resource obtained from base station l by a vehicle of priority Q.

Let the unit cost of spectrum resources be $R_{l}$, and let the seller cost be Y. If there are currently A vehicles that need to purchase spectrum resources, then the seller base station’s total cost is $Y_{1}^{l}+Y_{2}^{l}+\ldots +Y_{A}^{l}$. Therefore, the base station cost for each vehicle’s spectrum purchase and the seller base station’s total cost can be expressed as:

(2) $$Y_{i}^{l}=R_{l}V_{i}^{l}$$

(3) $$C_{l}=\sum_{i=1}^{A}R_{l}V_{i}^{l}$$

where *C_l_* denotes the total cost of the seller.

Since each buyer vehicle needs to pay the seller base station when purchasing spectrum resources, the resource cost of each buyer vehicle needs to be considered. The unit spectrum purchase price of the base station is $pr^{l}$. The purchase price of vehicles with different priorities and reputation values is different, so the spectrum purchase unit price and resource cost of vehicles are as follows:

(4) $$C_{i,Q}^{l}=\frac{pr^{l}}{\delta_{i}^{l}\cdot G_{Q}}$$

(5) $$T_{i,Q}^{l}=\sum_{l=1}^{K}V_{i}^{l}C_{i,Q}^{l}$$

where $C_{i,Q}^{l}$ represents the actual unit spectrum purchase price for different vehicles, $T_{i}^{l}$ represents the resource purchase cost of the vehicle, and $G_{Q}$ is the urgency assessment index of the vehicle. The seller base station obtains revenue while selling spectrum resources, and can obtain better revenue when the base station serves high-priority vehicles. Assuming that A vehicle needs to buy spectrum, the total revenue of the seller’s base station can be expressed as:

(6) $$U_{l}=\sum_{i=1}^{A}\left[\frac{pr^{l}\cdot V_{i}^{l}}{\delta_{i}^{l}\cdot G_{Q}}-\lambda \frac{{\left(V_{i}^{l}\right)}^{2}}{{\left(\delta_{i}^{l}\cdot G_{Q}\right)}^{2}}\right]$$

In the formula, λ is the risk coefficient. Since low-reputation or low-priority vehicles often require more complex verification processes in the consensus process, thus consuming more system computing power and resources, this risk term is introduced, which will make the seller rationally reduce the amount of resources supplied to them. For different buyer vehicles, its utility is related to its demand urgency, reputation value, and the amount of spectrum resources purchased, so the revenue reflected by the vehicle can be expressed as:

(7) $$L_{i}=\delta_{i}^{l}G_{Q}\sum_{l=1}^{K}\log\left(1+\varepsilon_{i}V_{i}^{l}\right)$$

where $\varepsilon_{i}\in [0,1]$ is the channel gain of the buyer’s vehicle. To sum up, the profit function of the buyer and the seller is as follows:

(8) $$E_{i}=L_{i}-T_{i,Q}^{l}=\delta_{i}^{l}G_{Q}\sum_{l=1}^{K}\log\left(1+\varepsilon_{i}V_{i}^{l}\right)-\sum_{l=1}^{K}V_{i}^{l}C_{i,Q}^{l}$$

(9) $$E_{l}=U_{l}-C_{l}=\sum_{i=1}^{A}\left[\frac{pr^{l}\cdot V_{i}^{l}}{\delta_{i}^{l}\cdot G_{Q}}-\lambda \frac{{\left(V_{i}^{l}\right)}^{2}}{{\left(\delta_{i}^{l}\cdot G_{Q}\right)}^{2}}\right]-\sum_{i=1}^{A}R_{l}\cdot V_{i}^{l}$$

In the formula, $E_{i}$ is the profit of the buyer’s vehicle, and $E_{l}$ is the profit of the seller’s base station, our goal is to maximize profits for both parties. At the same time, $V_{i}^{l}$ should be carried out under the condition of satisfying the restriction condition (1).

### 4.2. MLMF Stackelberg Game Model

The Stackelberg game (MLMF) is a strategy game involving leaders and followers who compete for a resource, ultimately maximizing their own interests. This game theory framework is adopted because of its inherent applicability to the hierarchical decision-making structure of vehicular network spectrum trading. First, in the proposed market model, base stations (BSs), as the primary spectrum resource holders, possess pricing power and naturally play the role of “leader.” Conversely, vehicles, as resource consumers, optimize their demand strategies based on observed prices, acting as “followers.” Second, unlike simultaneous-action games (such as Cournot games), the Stackelberg model’s sequential interaction mechanism accurately reflects the dynamic process of “pricing-response” in spectrum auctions, enabling leaders to predict follower reactions and formulate globally optimal strategies. Finally, the model effectively resolves the conflict between BS profit maximization and vehicle utility maximization.

In this paper, the selling base station is designated as the leader. By analyzing factors such as operating costs and profit targets, an initial price is set for each unit of spectrum. The price is then gradually adjusted based on the game process, playing a dominant role in pricing with the goal of increasing revenue from spectrum sales. Furthermore, we define the buyer vehicle as a follower, which responds with an appropriate amount of spectrum request based on its own communication needs and the pricing offered by the leader, with the goal of reducing the cost of purchasing spectrum while ensuring communication quality. This problem can be formulated as follows.

Leader spectrum pricing:

(10) $$\max_{pr\geq 0}\;E_{l}(pr,V)$$

(11) $$s.t.\;\sum_{N_{Q}}V_{i,Q}^{l}\leq P^{l}-\left(\sum_{N_{Q-1}}V_{i,Q-1}^{l}+\sum_{N_{Q-2}}V_{i,Q-2}^{l}+\cdots +\sum_{N_{1}}V_{i,1}^{l}\right)$$

Follower spectrum purchases:

(12) $$\max_{V\geq 0}\;E_{i}(V,pr)$$

where $E_{i}$ and $E_{l}$ are defined in (8) and (9), respectively.

The goal of the game is to find the equilibrium point (SE) of the game, in which the strategies of the leader and the follower do not change; that is, the maximization of the interests of both sides is achieved. SE can be defined as follows:

(13) $$E_{l}(pr^{*},V^{*})\geq E_{l}(pr,V^{*})$$

(14) $$E_{i}(V_{i}^{*},pr^{*})\geq E_{i}(V_{i},pr^{*})$$

where $pr^{*}$ is the solution of the seller’s base station spectrum pricing problem, and $V^{*}$ is the solution to the buyer’s spectrum purchase problem. Thus, ($pr^{*}$, $V^{*}$) is the SE of the Stackelberg game. The complete flow of the Stackelberg game is as follows:

- The seller’s base station sets the price of the unit spectrum based on the comprehensive consideration of its own remaining spectrum and operating costs.
- The buyer responds with the amount of spectrum required to be purchased based on the initial price of the spectrum in combination with its own spectrum demand.
- The buyer and the seller calculate their own profits based on the mutual information, and evaluate whether they can improve their own profits by changing their strategies. If there is a better strategy, the buyer and the seller make their own adjustments and return to the first step.
- When both sides have no better strategy to improve their own returns, it is regarded as reaching SE and the game is over.

Our equilibrium existence proof for the Stackelberg game is described in detail in Appendix A.

## 5. Consensus Plan

The core objective of the PhDPoR consensus mechanism is to construct a consensus framework possessing both security and reliability within the IoV environment, while remaining simultaneously committed to ensuring the fairness of node participation. The scheme deeply integrates the technical advantages of the practical Byzantine fault tolerance mechanism and establishes a strict block confirmation threshold; specifically, a block can only be recorded on the chain when more than two-thirds of the verification nodes reach a consensus. This mechanism effectively defends against potential security threats such as double-spending and Sybil attacks. The hierarchical architecture of PhDPoR is primarily characterized by a hybrid voting power allocation mechanism based on vehicle priority and reputation values. Compared to single-dimensional consensus schemes relying solely on reputation values, this design is more comprehensive. The remainder of this section will elaborate on voting power declaration, node reputation calculation, validator node selection, block verification, and the consensus process.

### 5.1. Calculation of Voting Rights

We combine historical and current reputation to form the final reputation assessment of the vehicle. Denote by $\delta_{Q}(t)=\omega \delta_{Q}(t-1)+\delta_{Q}^{*}(t)$, where ω∈(0,1) represents the weight of the reputation value of the previous vehicle on the reputation evaluation, and $\delta_{Q}^{*}(t)$ represents the latest reputation value, which is evaluated by the success of the transaction and the sincerity of the transaction process. $\delta_{Q}^{*}(t)$ is calculated as follows:

(15) $$\delta_{Q,i,l}^{*}(t)=\left\{\begin{array}{ll} \frac{V_{i}^{l}(t)}{\sum_{l=1}^{K}V_{i}^{l}(t)}, & \mathrm{honest}\\ -1, & \mathrm{dishonest} \end{array}\right.$$

To prevent unlimited growth of the reputation values of well-behaved vehicles, we use the Gompertz function to map the final reputation values to the interval [0,s) as follows:

(16) $$\delta (t)=s\cdot e^{-z\cdot e^{-u\cdot \delta_{Q}(t)}}$$

where s is the upper limit of reputation, z is the growth rate adjustment factor, and u is the reputation sensitivity factor.

In order to further optimize the security of the interaction process, we map the vehicle priority and its reputation value to the vehicle voting right based on the vehicle priority division and the vehicle reputation evaluation. The voting rights are calculated as follows:

(17) $$Power_{i\to j}=\frac{Q_{i}\cdot \delta_{i}}{\sum Q_{i}\cdot \delta_{i}}$$

where $Q_{i}$ represents the vehicle priority and $\delta_{i}$ represents the vehicle reputation value.

### 5.2. Node Reputation Calculation

We use a three-weight subjective logic model to calculate the reputation value of the base station, which takes into account the factors such as interaction weight allocation and timeliness, and ensures the security of the consensus process. The final reputation Rei,lend consists of subjective and objective opinions. The subjective opinion is the evaluation of the vehicle i to the base station l in the communication process. If the data given by the base station to the vehicle is useful, the vehicle will give a positive evaluation. The objective opinion is the evaluation of the base station based on the comprehensive consideration of the voting rights and subjective opinions of other vehicles. As shown in the Formula (18), where tr, un and di respectively represent the three subjective opinions of trust, uncertainty and distrust of the vehicle, $\rho_{i\to l}$ represents the interaction frequency, and the objective opinion of the vehicle is as follows:

(18) $$\left\{\begin{array}{l} tr_{i,l}^{obj}=\frac{\sum_{i=1}^{A}Power_{i,l}\cdot tr_{i,l}\cdot \rho_{i,l}}{\rho_{i\to l}}\\ un_{i,l}^{obj}=\frac{\sum_{i=1}^{A}Power_{i,l}\cdot un_{i,l}\cdot \rho_{i,l}}{\rho_{i\to l}}\\ di_{i,l}^{obj}=\frac{\sum_{i=1}^{A}Power_{i,l}\cdot di_{i,l}\cdot \rho_{i,l}}{\rho_{i\to l}} \end{array}\right.$$

The objective reputation assessment can be expressed as follows:

(19) $$Re_{i,l}^{obj}=tr_{i,l}^{obj}+\varphi \cdot un_{i,l}^{obj}$$

where φ∈[0,1] represents the influence of the uncertain behavior of the vehicle on the reputation. The final opinion of the vehicle is expressed as follows:

(20) $$\left\{\begin{array}{l} tr_{i,l}^{end}=\frac{tr_{i,l}^{sub}\cdot un_{i,l}^{obj}+tr_{i,l}^{obj}\cdot un_{i,l}^{sub}}{un_{i,l}^{sub}+un_{i,l}^{obj}-un_{i,l}^{sub}\cdot un_{i,l}^{obj}}\\ un_{i,l}^{end}=\frac{un_{i,l}^{sub}\cdot un_{i,l}^{obj}}{un_{i,l}^{sub}+un_{i,l}^{obj}-un_{i,l}^{sub}\cdot un_{i,l}^{obj}}\\ di_{i,l}^{end}=\frac{di_{i,l}^{sub}\cdot un_{i,l}^{obj}+di_{i,l}^{obj}\cdot un_{i,l}^{sub}}{un_{i,l}^{sub}+un_{i,l}^{obj}-un_{i,l}^{sub}\cdot un_{i,l}^{obj}} \end{array}\right.$$

where $tr_{i,l}^{sub}$, $un_{i,l}^{sub}$, and $di_{i,l}^{sub}$ respectively represent the subjective opinions of the vehicle itself, and the final reputation results can be obtained as follows:

(21) $$Re_{i,l}^{end}=tr_{i,l}^{end}+\varphi \cdot un_{i,l}^{end}$$

### 5.3. Block Message Storage

**Figure 2:**
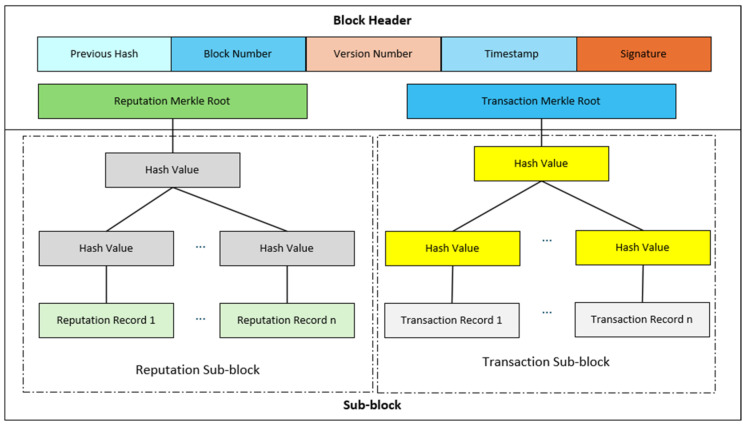
Block structure.

The data structure of the block we created is shown in Figure 2. The block consists of a block header and a sub-block. The block header includes the hash value, block number, version number, timestamp, block signature, reputation Merkle root, and transaction Merkle root of the previous block. The sub-block includes the reputation sub-block and transaction sub-block. The reputation sub-block contains the reputation voting and verification information to verify the legitimacy of the new additional block, and the reputation sub-block forms a Merkle tree. The transaction sub-block contains the data of the transaction market involved in the resource allocation process, and the transaction sub-block forms a Merkle tree. When a buyer sends an energy purchase request to the blockchain, the transaction is created and the block generation begins. When the request is sent to the IoV system, the verifier master node stores the request in the transaction pool and then begins the consensus process.

### 5.4. Reputation-Based Verifier Selection

This paper adopts a consortium blockchain architecture to achieve network-wide consensus by executing transaction verification on a set of pre-selected nodes. Within each time slot, the system independently ranks vehicles and base stations based on reputation scores. The top three-quarters of vehicles are selected as validator nodes. Simultaneously, the base station with the highest reputation value is designated as the primary node (leader), while the one with the second-highest reputation serves as the backup primary node, designed to assume the leader’s functions to maintain system operation in the event of a primary node failure. The primary node is responsible for constructing new blocks and broadcasting them to the group of validator nodes. Upon receiving the blocks, the validator nodes verify the data; once the number of verifying nodes exceeds the two-thirds threshold, the block is formally appended to the blockchain ledger.

### 5.5. PhDPoR Consensue Process

After determining the optimal pricing and resource allocation strategy, the transaction between buyers and sellers will reach a consensus through the alliance blockchain. The algorithm flow we proposed is as follows:

1. Leader and verifier election: Legitimate authorized entities are ranked by reputation value, M vehicles are selected as validators, and the base station with the highest reputation value is selected as the leader of this round of consensus to generate a block and broadcast the block to other validators.

2. Block verification: The verifier node verifies the received new block. If it finds that the priority declared by the vehicle is inconsistent with its real priority, it will be marked as a malicious node and the reputation value will be reduced; specifically, this behavior is verified by cross-referencing the priority of a claim with observable data behavior recorded on the ledger. The authenticated node generates an audit triple containing <id,h,s> and broadcasts it to other nodes, where id represents the block number, h represents the block hash value, and s is the signature of the node.

3. Audit decision: If the audit result received is greater than $\frac{\left(M-1\right)}{3}+1$, then each node compares with the others and sends an <id,h,s> agreement message to the leader. If the leader receives an agreement message from more than $\frac{2(M-1)}{3}+1$ nodes, the leader appends the block to the blockchain and broadcasts it to all nodes, and at the same time, the leader requests the node that sent the disagreement message to check the audit again.

4. Reputation update: After each round of consensus, the node reputation is dynamically adjusted. If no consensus is reached at the end of the time, the consensus of this round will be abandoned.

RSUs participate in the verification process as auxiliary consensus nodes, which independently verify the accuracy of blocks and record transaction data to enhance the reliability of the system.

### 5.6. Threat and Security Analysis

In an IoV environment, both vehicles and base stations are potential targets. Firstly, malicious vehicle behavior can be categorized into information deception and malicious defamation. This involves sending false information to other vehicles to reduce traffic efficiency or providing unrealistic feedback to base stations to denigrate or praise them. Secondly, although base stations are subject to oversight by relevant authorities, they still face the risk of intrusion. However, due to the limited number and capabilities of attackers, large-scale intrusions into vehicles and base stations are unrealistic. Therefore, this study reasonably assumes that most vehicles are honest and a small number of base stations are at risk of being compromised. Intruded base stations will gain access to their stored information.

In the proposed model, if a consensus node discovers that the information uploaded by certain vehicles is inconsistent with that of nearby honest vehicles, it lowers the reputation value of those vehicles, reducing their voting power in resource allocation. Similarly, compromised base stations will also receive low reputation ratings from honest nodes due to their malicious behavior, reducing their consensus participation and thus inhibiting their impact on system resource allocation. These malicious behaviors will create significant disadvantages for nodes in future resource allocation processes; therefore, malicious nodes must carefully consider whether they can bear the costs.

### 5.7. Total Computing Power Consumption

We model the system’s total computational energy consumption $B_{total}^{comp}$ as the cumulative sum of energy consumption during the resource allocation phase and the consensus phase:

(22) $$B_{total}^{comp}=B_{alloc}^{comp}+B_{cons}^{comp}$$

- Energy consumption during the resource allocation phase: This phase involves the iterative convergence of the game, where H represents the number of iterations required to reach equilibrium. The total energy is the sum of the computational costs of the base station and all i vehicles:
  (23) $$B_{alloc}^{comp}=H\cdot (l\cdot B_{BS}+i\cdot B_{veh})$$

The vehicle’s optimal strategy can be obtained through a closed-form solution.

- Consensus Phase Energy Consumption: This phase consumes resources for reputation calculation, voting power calculation, and block verification. The energy consumption for M validators is:
  (24) $$B_{cons}^{comp}=\sum_{m=1}^{M_{val}}\left[\kappa \cdot F^{2}(D_{rep}+D_{wei}+D_{ver})\right]$$
  where κ is the effective switched capacitor, F is the processor frequency, and $D_{rep}$, $D_{wei}$, and $D_{ver}$ represent the number of CPU cycles for reputation calculation, voting power calculation, and block verification, respectively.

### 5.8. Time Delay

In the proposed scheme, the total latency of spectrum allocation consists of spectrum allocation time and consensus time:

(25) $$L_{total}=L_{alloc}+L_{cons}$$

- Spectrum allocation time: The allocation phase is based on an MLMF Stackelberg game, which involves iterative interactions between the base station and the vehicle. Let H be the number of iterations required for the game to reach a Stackelberg equilibrium (SE). In each iteration, the time consumption consists of data transmission latency and computation latency, as detailed below:
  (26) $$L_{alloc}=\sum_{h=1}^{H}\left(\frac{l\cdot W_{price}}{X_{dl}}+\frac{i\cdot W_{req}}{X_{ul}}+L_{comp}^{BS}+L_{comp}^{veh}\right)$$
  where $X_{dl}$ and $X_{ul}$ are the average downlink and uplink data transmission rates, respectively; $W_{price}$ and $W_{req}$ are the data sizes of the price broadcast message and bandwidth request message, respectively; and $L_{comp}^{BS}$ and $L_{comp}^{veh}$ are the computation times for the base station and the vehicle, respectively.
- Consensus Phase Time: The consensus delay in the PhDPoR mechanism mainly stems from block propagation and the voting process among validators; let $W_{block}$ be the block size and $M_{val}$ be the number of validators. The consensus delay is as follows:
  (27) $$L_{cons}=\frac{W_{block}}{X_{dl}}+\frac{N_{tx}\cdot C_{ver}}{F}+\frac{M_{val}\cdot W_{sig}}{X_{ul}}$$
  where $C_{ver}$ is the number of CPU clock cycles required to verify a single digital signature, $N_{tx}$ is the number of transactions in the block, and $W_{sig}$ is the size of the voting signature message.

## 6. Experimental Simulation

### 6.1. Simulation Environment

The simulation scenario in this paper is simulated by MATLAB 2022b on a computer with i9-13900HX 2.20 GHz, 16 GB RAM running Windows 11. We compare the proposed scheme with the (PoW, PoR, PBFT) benchmark schemes to evaluate our scheme. This simulation considers standard parameters to primarily evaluate system performance such as latency, benefit, game effectiveness, and reputation impact. In the V2I network, the vehicle and the base station are connected by a wireless link, and the spectrum allocation task uses an optimal allocation method integrating PBFT, priority, and reputation values. In the simulation scenario, the transmission radius of the base station is 5 km, the transmission radius of the RSU is 300 m, the moving speed of the vehicle is 60–75 km/h, and the number of vehicles is 20–120. Other specific simulation parameters are shown in Table 3.

### 6.2. Simulation Result

This section compares the proposed scheme with PoW, PBFT, and PoR in terms of transaction latency and throughput under different vehicle densities, evaluates its scalability, and assesses PhDPoR in terms of utility optimization and security.

#### 6.2.1. Performance Evaluation

**Figure 3:**
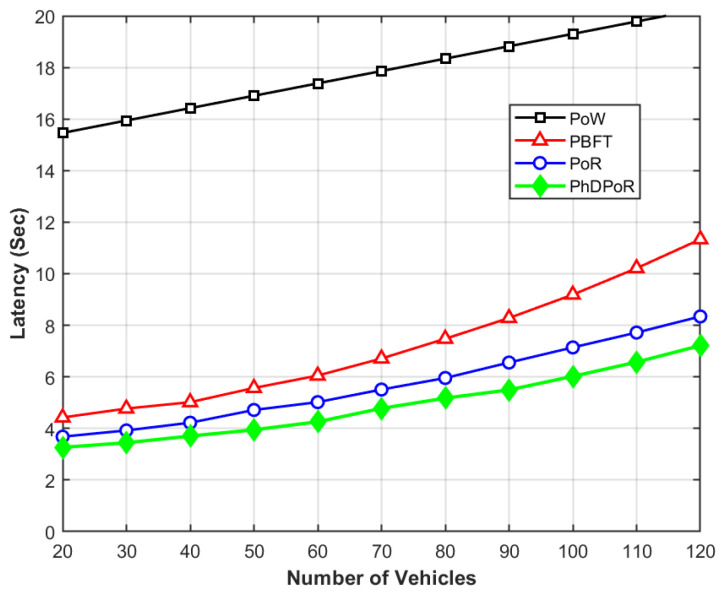
Comparison of latency with the number of vehicles.

**Figure 4:**
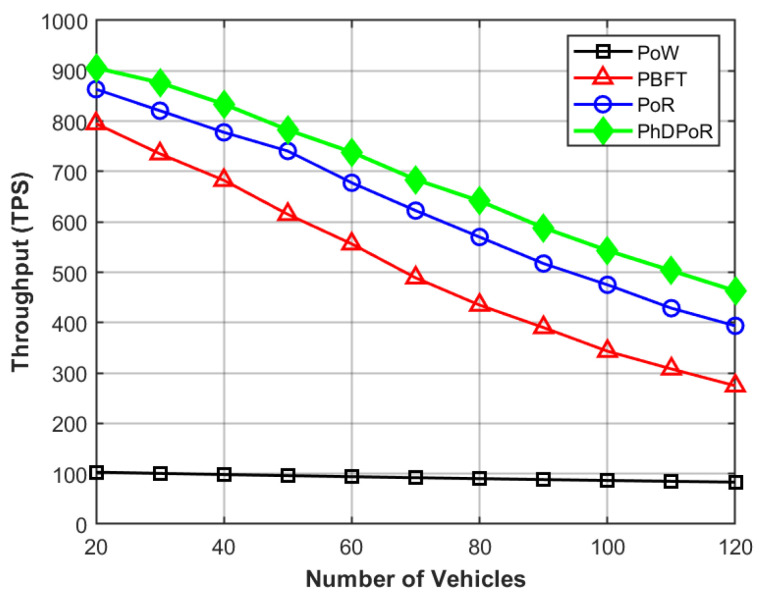
Comparison of throughput with the number of vehicles.

The latency we explore represents the time difference between the blockchain receiving a resource request and the buyer receiving a response, as shown in Figure 3. As the number of vehicles in the network increases, the latency of the PhDPoR consensus mechanism is significantly lower than other benchmark algorithms. Figure 4 compares the number of transactions processed by nodes in the network, demonstrating that even with increasing network congestion, the proposed scheme maintains higher throughput compared to benchmark algorithms.

Scalability can be measured by throughput and latency. As shown in Figure 3 and Figure 4, when the number of vehicles increases from 20 to 120, the growth and decline trends of PhDPoR are superior to those of the PoW, PoR, and PBFT algorithms. This clearly demonstrates that PhDPoR has good scalability.

#### 6.2.2. Equilibrium Evaluation of MLMF Stackelberg Games

**Figure 5:**
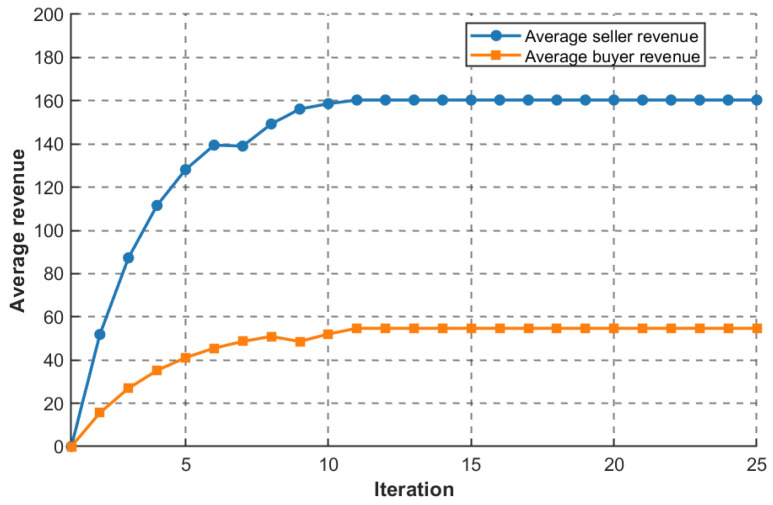
The relationship between the income of both sides of the game and the number of iterations.

Assuming that the bandwidth provided by each base station is $\{P^{l}\}\in [20,50]Mhz$, we verify the convergence of the MLMF Stackelberg game by observing the average income of buyers and sellers. As shown in Figure 5, the income of buyers and sellers increases gradually, reaches the maximum at about the 11th iteration, and then does not change. At this point, the game is considered to reach Stackelberg equilibrium.

#### 6.2.3. Validity of Priority and Reputation Values

**Figure 6:**
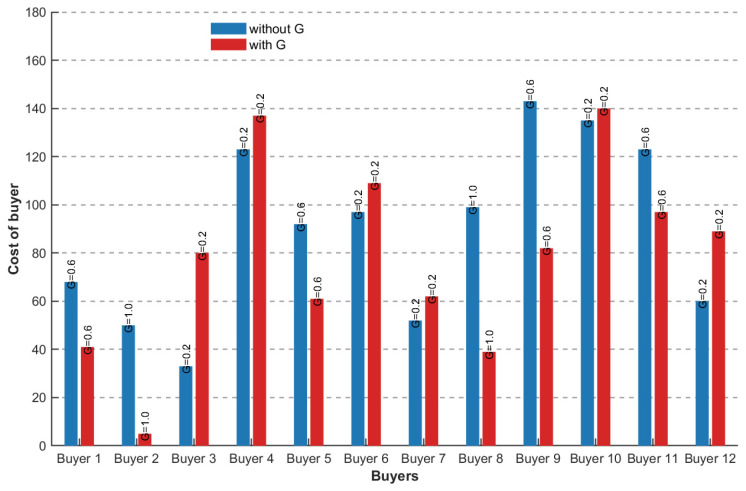
The relationship between the buyer’s cost and G.

In this paper, we introduce the urgency evaluation index G of vehicles with different priorities, randomly select 12 buyer vehicles, and observe the cost change in the urgency evaluation index G, as shown in Figure 6, where vehicles 2 and 8 are the vehicles with the highest priority, before the introduction of G, vehicle 8 needs a higher cost, and vehicle 3 needs a very low cost, but after the introduction of G. There is a significant change in cost between high priority and low priority vehicles. Experimental results confirm that vehicles with high urgency possess a significant advantage in the resource bidding process, thereby enabling the effective acquisition of high-quality spectrum resources.

**Figure 7:**
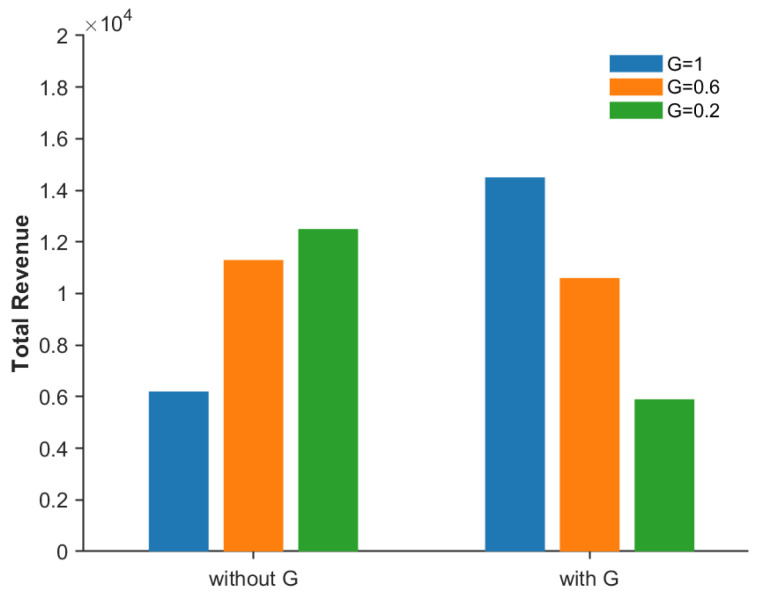
The relationship between the seller’s revenue and G.

To verify the impact of introducing vehicle demand urgency levels and the effects of vehicles with different urgency levels on seller base station revenue, we compared the revenue changes in seller base stations from vehicles of different priorities before and after setting the vehicle urgency level index. As shown in Figure 7, the vertical axis represents the total revenue of the base station, and the horizontal axis represents the revenue brought by different vehicles before and after introducing the urgency level index. Specifically, after introducing G, the revenue share of vehicles with an urgency level of 1 increased significantly, attributed to the optimization of the allocation mechanism. Compared with the resource mismatch caused before, the introduction of G can accurately identify the marginal utility advantage of high-priority vehicles in the game phase and adaptively allocate the optimal bandwidth that maximizes net revenue to them, thereby obtaining the potential profit of high-value nodes. Therefore, seller base stations will tend to prioritize serving high-priority buyers, thereby improving the social benefits under spectrum resource allocation.

To evaluate the specific impact of the reputation mechanism on vehicle transaction costs, eight buyer vehicles were randomly selected in the experiment, and the cost variations before and after the introduction of reputation evaluation were compared, as shown in Table 4. In the experimental setup, vehicle 4 was designated as a low-reputation node, while vehicle 5 was configured as a malicious node with an extremely low reputation value. The results indicate that after the implementation of the reputation mechanism, the transaction cost of the malicious vehicle exhibited a significant increasing trend; although the cost of vehicle 4 rose, the magnitude of the increase was significantly lower than that of vehicle 5; in contrast, the costs of other normal vehicles showed no significant fluctuations. The results confirm that the reputation mechanism can effectively suppress the system impact of malicious vehicles by implementing a strict cost penalty strategy without affecting normal vehicles, thereby effectively ensuring the safe and stable operation of the system.

#### 6.2.4. Utility Optimization and Safety Assessment

**Figure 8:**
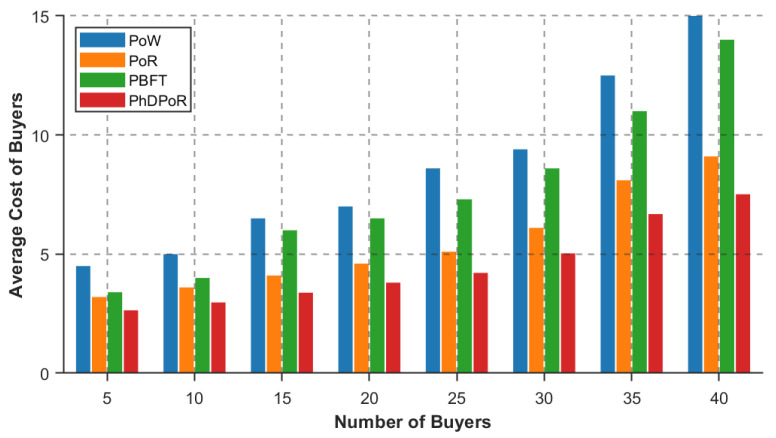
Comparison of buyer’s average cost for different number of buyers and different algorithms.

**Figure 9:**
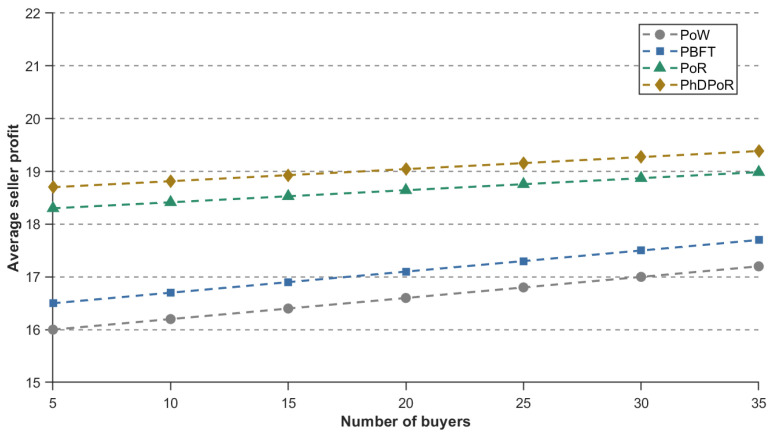
Comparison of the average profit of sellers with different number of buyers and different algorithms.

To validate the effectiveness of the PhDPoR algorithm, this paper conducts a comparative analysis against the PoW, PoR, and PBFT algorithms. Figure 8 and Figure 9 respectively illustrate the impact trends of each algorithm on buyer vehicle costs and seller base station returns as the scale of buyer nodes increases. Specifically, Figure 8 indicates that while buyer costs under all algorithms exhibit an upward trend with the increase in the number of buyers, the PhDPoR algorithm outperforms PoW, PoR, and PBFT in terms of cost control. Meanwhile, data in Figure 9 demonstrates that the proposed algorithm yields higher returns for seller base stations compared to the baseline algorithms. These results indicate that PhDPoR effectively achieves the joint optimization of economic benefits for both buyers and sellers during the spectrum allocation process.

**Figure 10:**
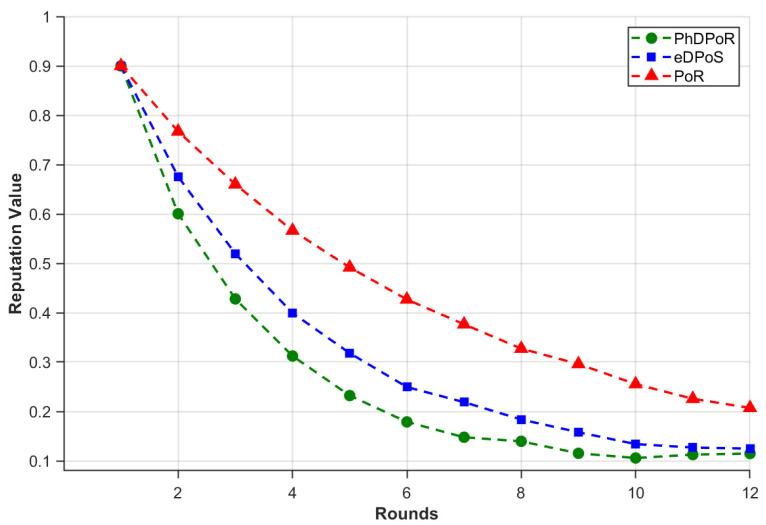
Change in reputation value of malicious nodes under different algorithms.

Malicious nodes are bound to appear in the open Internet of Vehicles environment. The scheme proposed in this paper is to calculate the voting rights according to the vehicle priority and reputation value. In order to verify the effect of the PhDPoR algorithm on malicious nodes, we compare it with eDPos and PoR, focusing on the change in reputation value of each algorithm on malicious nodes. Malicious nodes will give negative evaluation to normal vehicles and interfere with the Internet of Vehicles. Specifically, as shown in Figure 10, under the same number of rounds, the reputation penalty of PhDPoR for malicious nodes is more aggressive. Since the nodes are positively correlated with the reputation, the influence of malicious nodes converges rapidly, and the interference of malicious nodes on the consensus process is reduced, thus improving the robustness and security of the system.

To sum up, the scheme proposed in this paper adjusts the allocation of market resources, improves social benefits, and optimizes the profits of buyers and sellers. In addition, the reputation-based voting method can effectively detect changes in the reputation values of malicious nodes, thereby enhancing the security of the system.

## 7. Conclusions and Prospect

This paper proposes a PhDPoR-based consensus mechanism to optimize spectrum resource allocation and enhance security in vehicular networks. We construct a consortium blockchain framework integrating smart contracts to ensure the immutability and traceability of stored transactions. Based on this, we design an MLMF Stackelberg game model to maximize the economic utility of both buyers and sellers, and employ an asymmetric pricing strategy to dynamically adjust resource allocation based on vehicle priority and reputation. Simulation results demonstrate that the scheme effectively balances computational overhead and system performance. Specifically, PhDPoR can effectively adjust resource allocation, improve overall social benefits, and maintain good latency and throughput in high-vehicle-density scenarios. Furthermore, this scheme outperforms the eDPoS and PoR algorithms in handling malicious nodes, ensuring system security.

In the future, we will focus on further optimizing the algorithm to enhance its adaptability in highly heterogeneous networks. In addition, we plan to explore the integrated application of cryptographic technologies to further strengthen privacy protection while maintaining system efficiency.

This research validates the effectiveness of the proposed spectrum resource allocation scheme and provides valuable reference for resource management in future smart city infrastructure.

## Figures and Tables

**Table 1:** Vehicle demand classification and priority mapping index.

Vehicle Purpose	Demand Type	Vehicle Examples	Social Impact	Priority Level	Urgency Index Value
Special Purpose	Emergency	Ambulance, fire truck, police car	Emergency Safety	1	1.0
Special Purpose	General	Tanker, tow truck	Special Needs	2	0.6
Public Transport	Logistics/Public Transit	Express truck, logistics trailer, bus, tour bus	Social Transport	3	0.6
Private/Sedan	Commercial/Private	Rideshare car, taxi, hitchhiking car, private car	General Transport	4	0.2

**Table 2:** Summary of main functional modules and interfaces.

Module Name	Trigger Condition	Input Parameters	Description
InitGame	Vehicle Request	RequestMsg	Initializes game
TriggerConsensus	Equilibrium Reached	$\mathrm{GameResult}\;\left(pr^{*},V^{*}\right)$	Proposes a new block
ExecuteAllocation	Consensus > 2/3	BlockData	Executes transfer
UpdateReputation	Audit Completed	AuditResult	Updates reputation

**Table 3:** Simulation parameters.

Parameter	Value
Blockchain type	Alliance Blockchain
Number of base stations	4
Number of RSUs	8
Number of vehicles	[20–120]
Base station transmission radius	5 km
RSU transmission radius	300 m
Speed of vehicle movement	[60–75] km/h
Vehicle priority	[1, 4]
Block size	1.0 MB
Block propagation delay	0.42 S
Noise power	−174 dBm/Hz
Transmission power	1.2 W

**Table 4:** Influence of reputation value on buyer’s cost.

	Buyer 1	Buyer 2	Buyer 3	Buyer 4	Buyer 5	Buyer 6	Buyer 7	Buyer 8
Without reputation	115	220	140	180	110	136	210	215
With reputation	120	195	143	255	305	126	215	207

## Data Availability

Data are contained within the article.

## Appendix A

The profit functions for both the buyer and seller are shown in Formulas (8) and (9) in the main text. To verify the existence of SE in the MLMF Stackelberg game, for any pr>0, we obtain the first derivative of the buyer’s profit function as:

$$\frac{\partial E_{i}}{\partial V_{i}^{l}}=\frac{\delta_{i}^{l}\cdot G_{Q}\cdot \varepsilon_{i}}{1+\varepsilon_{i}\cdot V_{i}^{l}}-\frac{pr^{l}}{\delta_{i}^{l}\cdot G_{Q}}$$

Setting the first derivative to 0, we obtain the vehicle’s unique optimal response function $V^{*}(pr)=\frac{{\left(\delta_{i}^{l}\cdot G_{Q}\right)}^{2}\cdot \varepsilon_{i}}{pr^{l}}-\frac{1}{\varepsilon_{i}}$. To verify the properties of this solution, the second derivative with respect to $E_{i}$ is:

$$\frac{\partial^{2}E_{i}}{\partial {\left(V_{i}^{l}\right)}^{2}}=-\frac{\delta_{i}^{l}\cdot G_{Q}\cdot \varepsilon_{i}^{2}}{{\left(1+\varepsilon_{i}V_{i}^{l}\right)}^{2}}\leq 0$$

Therefore, $E_{i}$ is a strictly concave function of $V_{i}^{l}$, and thus there exists an optimal and unique response $V^{*}$.

The seller’s base station aims to maximize its profit $E_{l}$. To prove the uniqueness of the equilibrium, we substitute $V^{*}$ into $E_{l}$ and take the second derivative with respect to the price pr. After simplification and differentiation, the second derivative of the base station’s profit is:

$$\frac{\partial^{2}E_{l}}{\partial {\left(pr^{l}\right)}^{2}}=\sum_{i=1}^{A}\left[-\frac{6\lambda K_{i}^{2}}{{\left(\delta_{i}^{l}G_{Q}\right)}^{2}\cdot {\left(pr^{l}\right)}^{4}}-\frac{2R_{l}K_{i}}{{\left(pr^{l}\right)}^{3}}\right]\leq 0$$

where $K_{i}$ is a constant coefficient. Since the second derivative is less than 0, it indicates that the base station’s profit function $E_{l}$ is a strictly concave function with respect to price. According to convex optimization theory, a strictly concave function must have a unique global maximum within the feasible region. Therefore, this MLMF Stackelberg game model has a unique equilibrium solution (SE), namely $\left(pr^{*},V^{*}\right)$.
